# Ub-ISAP: a streamlined UNIX pipeline for mining unique viral vector integration sites from next generation sequencing data

**DOI:** 10.1186/s12859-017-1719-4

**Published:** 2017-06-17

**Authors:** Atul Kamboj, Claus V. Hallwirth, Ian E. Alexander, Geoffrey B. McCowage, Belinda Kramer

**Affiliations:** 1 Children’s Cancer Research Unit, Kids’ Research Institute, The Children’s Hospital at Westmead, Locked Bag 4001, Westmead, NSW 2145 Australia; 2 Gene Therapy Research Unit, Children’s Medical Research Institute and The Children’s Hospital at Westmead, Westmead, NSW Australia; 3 The University of Sydney, Discipline of Paediatrics and Child Health, Westmead, NSW Australia; 4 Cancer Centre for Children, The Children’s Hospital, Westmead, NSW Australia

**Keywords:** Gene therapy, Integration site analysis, Next-generation sequencing, Viral vectors

## Abstract

**Background:**

The analysis of viral vector genomic integration sites is an important component in assessing the safety and efficiency of patient treatment using gene therapy. Alongside this clinical application, integration site identification is a key step in the genetic mapping of viral elements in mutagenesis screens that aim to elucidate gene function.

**Results:**

We have developed a UNIX-based vector integration site analysis pipeline (Ub-ISAP) that utilises a UNIX-based workflow for automated integration site identification and annotation of both single and paired-end sequencing reads. Reads that contain viral sequences of interest are selected and aligned to the host genome, and unique integration sites are then classified as transcription start site-proximal, intragenic or intergenic.

**Conclusion:**

Ub-ISAP provides a reliable and efficient pipeline to generate large datasets for assessing the safety and efficiency of integrating vectors in clinical settings, with broader applications in cancer research. Ub-ISAP is available as an open source software package at https://sourceforge.net/projects/ub-isap/.

## Background

The goal of many gene therapy strategies is to stably integrate new DNA sequences into the genome of therapeutically relevant target cell populations, and their progeny. Engineered viral vectors are capable of performing this function, either through their underlying biological properties, as in the case of retroviral vectors, or through combinatorial approaches whereby elements driving integration are incorporated into vector design and delivery, such as the piggyBac system for adeno-associated viral (AAV) vectors [1]. One important consequence of achieving stable integration of DNA cassettes into the target cell genome, however, is the possibility that the integration event will disrupt the function of surrounding gene sequences with unpredictable consequences [2]. The level of risk associated with any particular vector is related to its intrinsic integration pattern, and to a lesser extent, the transgene cassette cargo, and this characteristic of possible genotoxicity [3] has been best recognised following the use of ƴ-retroviral vectors to target the haematopoietic stem/progenitor cell (HSPC) of patients being treated for immune deficiencies. These vectors, in addition to lentiviral vectors, are the preferred choice for HSPC targeted gene therapy (GT) applications [4–6], and integrate in a semi-random fashion in the genome [7–10] with each unique integration site (IS) serving as a distinctive genetic identifier for the initial integration event in the vector-marked cells and their progeny [11]. Notably, this pattern of vector integration can result in the dysregulation of nearby genes, leading to malignant clonal expansion of a gene-modified cell population, in a phenomenon known as insertional mutagenesis (IM) [12, 13]. The first report of IM associated with retroviral integration events are those that occurred in two X-linked severe combined immunodeficiency (SCID-X1) patients who were treated with a ƴ-retroviral vector in a clinical trial, and who later developed a lymphoproliferative leukaemia [14]. Integration site (IS) analysis of the clonal leukaemic cell populations determined the identity of the dysregulated gene in both patients as the *LMO2* gene, leading to deviant expression of the LMO2 protein, a proto-oncogene implicated in the causation of T cell leukaemias [15]. Similar events occurring in subsequent patients treated with gene therapy for SCID-X1 have highlighted the importance of including IS analysis in assessing the safety of retroviral vectors [16].

The mapping of ISs to their genomic location is an important step in understanding the potential genotoxicity associated with the use of GT vectors in the clinic, in understanding the mechanisms of insertional mutagenesis (IM), and in enabling development of improved vectors in preclinical studies [16]. IS identification can also be used for retroviral mutagenesis screens, by identifying the genes adjoining integrated viral sequences as candidate cancer driver or progression genes [17]. In the most commonly used protocols, ISs are identified via amplification of vector-chromosome junction fragments, comprising both proviral and flanking cellular genome sequences, after ligation of DNA linkers of known sequence to provide for PCR primer binding. PCR amplicons spanning the vector/genome junction are then sequenced and aligned to the host genome to determine the genomic coordinates of the IS [18, 19]. The advent of next-generation sequencing (NGS) technology has greatly enhanced the depth of IS analysis datasets by producing millions of sequence reads from complex vector-chromosome junction fragment libraries. However, since individual ISs can be represented multiple times in NGS data owing to the amplification steps involved in library preparation, the mapping, identification and annotation of ISs constitutes a challenging bioinformatics task.

Although web-based tools are available for IS analysis using NGS data, problems arise when access becomes difficult for example, Quickmap tool, [20] or when online tools (for example SeqMap 2.0 (http://seqmap.compbio.iupui.edu/) no longer provide mapping to the most recent human genome assembly. For this reason, we developed a stand-alone and user-friendly UNIX-based IS analysis pipeline, Ub-ISAP [21]. In addition to availability that is independent of web-based programs, Ub-ISAP’s mapping process can be achieved with reference to the human (or other) genome assembly version of choice. Moreover, since web-based tools that are currently available for IS analysis are only designed to deal with single-end reads of junction fragments, Ub-ISAP was designed to accept both single-end and paired-end read sequence files for instances in which investigators seek to increase their confidence in determining IS identity. Ub-ISAP is a first-of-its-kind software available for IS analysis to accommodate paired-end read input. Our particular application entailed the requirement to analyse ISs in genomic DNA (gDNA) derived from patient samples generated as part of a Phase I gene therapy trial.

### Implementation

Ub-ISAP was designed to analyse junction fragments generated from a range of custom library preparation methods including LM-PCR [22] or Mu transposition-based methods [19] These methods use either restriction endonuclease (RE) digestion or Mu transposase treatment to fragment gDNA extracted from vector-transduced cells in order to ligate linkers of known sequence (in the case of RE digestion methods), or use the Mu transposon sequence, to prime subsequent PCR amplification of the junction fragments. Known vector sequences at the 5′ and 3′ outer limits of the integrated vector cassette (the terminal repeat region, TR) provide binding sites for the complementary PCR primers. Depending on the design of forward and reverse primers, PCR amplified fragment libraries can contain known vector 5′ TR sequence abutting adjacent gDNA upstream of the integration site, known 3′ TR vector sequence abutting adjacent gDNA downstream of the integration site, or contain both of these types of fragments. In addition, since both the 5′ and 3′ TR regions of an integrated vector cassette commonly contain stretches of identical sequence, the primers designed to amplify from the TR region of the cassette can bind to both the 5′ and 3′ end of the integrated cassette. As a result, up to half of the resultant amplicons generated by PCR will have been primed into the vector cassette, rather than into the adjacent gDNA. These amplicons do not provide any information regarding the co-ordinates of the integration site, and will fail to align to the query genomic sequence in the subsequent analysis.

For our work, fragment libraries generated using Mu transposase methodology, and using primers designed to amplify from the 3’TR of the integrated cassette into downstream gDNA were size-selected to yield fragments ranging from 100 to 400 bp in length. Re-amplification of the library fragments then facilitated the incorporation of sequencing platform-specific adaptors and an additional six-nucleotide sequence barcode to enable sample recognition for multiplexing. Junction fragment DNA libraries were sequenced using the Thermofisher Ion-torrent proton Personal Genome Machine (PGM). As described above, the sequencing reads comprised both the genomic sequence required for IS mapping and viral sequence for identification of candidate junction fragments.

### Selection of candidate junction fragments and trimming of vector sequences

Ub-ISAP accepts multiple raw NGS reads in a single FASTQ file (with .fastq file extension) for single reads or two FASTQ files (with .fastq file extensions) for paired-end reads, which can be selected on being prompted by the command line or as command line parameters. The only other inputs required from the user are the 5′ TR primer and the 3′ distal primer sequences used in library preparation. The command line parameters for processing of both single and paired end reads are as follows:

./Ub-ISAP.sh <1(Single) or 2 (Paired) > <Directory> <Indexedfile> <bedfile> <fastqfile1> <Forwardprimer> <ReversePrimer> <fastqfile2> <Forwardprimer> <ReversePrimer>

**Fig. 1:**
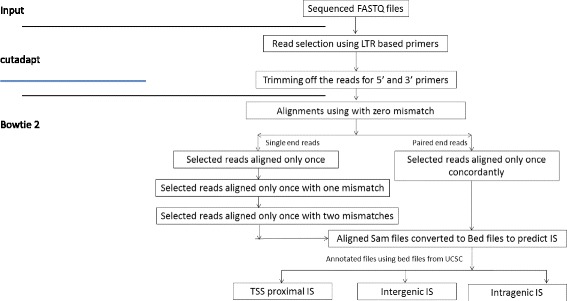
Ub-ISAP pipeline schematic diagram. Candidate reads having the TR-based primers are selected and primers were trimmed off both the 5′ and 3′ prime ends of selected reads. These reads are aligned against the reference host genome using Bowtie2 allowing no mismatches. The paired-end reads that align concordantly only at one location are selected for annotation, whereas single end reads that align only at one location are realigned twice allowing one and two mismatches respectively. The reads that align only at a single position after final alignment are selected for annotation. The unique reads are classified as TSS-proximal, intragenic and intergenic

The initial step, using the Python program cutadapt [23] takes the user-defined TR primer sequence to select candidate junction fragment reads, which are then trimmed to generate query sequences for alignment. This is achieved through a search for a sub-sequence consisting of the last 20 or more nucleotides of known TR sequence, allowing up to two mismatches for TR sequences of ≥20 bases, one mismatch for 10-19 bases and no mismatch below 10 bases. If the TR sequence is found, it is trimmed from the selected read. In the case of paired–end data, reads are selected only if 5′ primer sequences (TR sequence for read 1 and adaptor sequences for read 2) are identified in both sets of reads. All reads without the specified proximal sequence or sub-sequence are discarded. Since a proportion of the remaining candidate reads may contain the 3′ distal primer/adaptor sequence, or part thereof, this is trimmed where necessary to facilitate end to end alignment. Absence of this sequence does not render the read ineligible for inclusion, as individual read lengths (and therefore the likelihood of the 3′ primer sequence being included) are dependent on the size of fragments generated during library preparation, PCR amplification biases and NGS platform-specific variables. Lastly, trimmed reads smaller than 30 bp in length are eliminated prior to alignment [16], with only sequence reads having TR-chromosome junction and at least 30 bp in length being selected for further analysis (Fig. 1).

### Alignment

To identify the ISs within the host genome, trimmed reads are mapped to the reference genome using Bowtie2 with pre-defined filters. These pre-defined filters could be altered with basic understanding of Unix scripting. For our datasets, Ub-ISAP aligned the selected reads against the Genome Reference Consortium Human build 37 (GRCh37/hg19), which is set as the default genome file. Selection of an alternative genome (for example an updated human genome or another species) is possible at this point by selection of the “other” option in the software and provision of the appropriate filenames for the indexed genome and its associated bedfile. The bed file must be in the order of chromosome number, starting position, end position, gene accession number, gene symbol and strand (+/−).

The process for end-to-end alignment proceeds as follows:

1. Single end reads are aligned to the reference genome allowing zero mismatches and reads that align to more than one location are discarded.
2. Reads from step 1 are aligned again, this time allowing one mismatch. Any reads aligning at more than one genomic location are again discarded.
3. Reads from step 2 are aligned again, but allowing two mismatches. Any reads aligning at more than one genomic location are again discarded.

The reads that have aligned only at one location following three alignment rounds are considered for calling as Unique IS and subsequent annotation. This strategy is implemented to reduce false-positive mapping of reads containing one or two base position errors generated during the sequencing reaction [24]. For paired-end reads, sequences that align concordantly only at one position with zero mismatches are considered for calling as Unique IS with subsequent annotation. The condition for concordant alignment requires that a pair of reads has aligned with the expected relative mate orientation and expected range of distance between the mates. The nucleotide preceding the first position of every mapped forward orientation read is considered to denote the position of the respective integration event. Similarly, the last nucleotide position of every read mapped in reverse orientation is denoted as the position of integration [24]. These identified ISs are then processed to create the unique IS dataset (Fig. 1).

### Integration site annotations

Recent NGS-based IS analyses have demonstrated that different types of viral vectors have distinct integration patterns [16]. For example lentiviral vectors display a strong preference for intragenic regions whereas foamy viral vectors have a modest preference for Transcription Start Site (TSS)-proximal regions [16]. The tendency of γ-retroviral vectors to integrate in proximity to TSSs of genes can result in the subsequent dysregulation of gene expression and can also lead to targeting of proto-oncogenes in tumour initiation [25]. Since IS location is therefore of interest in elucidating vector integration behaviour, Ub-ISAP includes a final step for annotation of ISs as Transcription start site (TSS)-proximal (located within +/− 2.5 kB of a TSS), intragenic (located between the TSS and the transcription end site, but excluding those sites already classified as TSS-proximal) or intergenic (all remaining sites) relative to University of California Santa Cruz (UCSC) known genes. Proximity to nearby genes is determined with reference to the UCSC Known Genes database (July 2016) using BEDtools (http://bedtools.readthedocs.io/en/latest/).

### Software requirement

Ub-ISAP functions on LINUX command line and has been developed and tested on the UBUNTU operating system version 14.0. The required installed programs for running Ub-ISAP are cutadapt, Bowtie2, Bedtools and Perl.

## Results and discussion

Ub-ISAP provides researchers with a consistent methodology for the extraction of vector ISs from NGS data generated from gDNA (regardless of species origin) without the need to develop custom scripts. In addition to evaluating the computational performance of Ub-ISAP using datasets derived from complex junction fragment libraries (prepared as described above), we also tested Ub-ISAP against a web-based tool for IS analysis (VISA) [16], and also compared the output from Ub-ISAP against the output from two published studies for which the original raw sequence read files were available [1, 24].

### Computational performance of UB-ISAP on IS datasets from GT studies

The computation performance of Ub-ISAP was investigated using NGS data sets (Datasets 1 and 2, Table 1) generated from two independent sets of fragment libraries prepared from an immortalised human cell line transduced with a clinical grade γ-retroviral vector in our laboratory.

**Table 1:** Results of IS analysis of NGS datasets by Ub-ISAP

Sample	Single/Paired end reads	# Reads	Reads filtered	Reads aligned zero mismatch	Reads aligned one mismatch	Reads aligned two mismatches	Unique IS	TSS Proximal	Intragenic	Intergenic
1	Single	675,969	200,526	101,622	100,027	67,201	1981	391	793	797
2	Single	584,650	162,394	78,236	76,808	51,210	1789	399	631	756

These datasets initially contained 675,969 and 584,650 raw single end sequence reads, generated from the Thermofisher Ion Torrent PGM. TR sequence filtering using cutadapt yielded 85.4% and 84.4% of the initial input reads (datasets 1 and 2 respectively). Removal of reads less than 30 bp reduced the number of TR-selected and trimmed reads to 200,526 (29.7% of input for data set 1) and 162,394 (27.8% of input for dataset 2). Alignment to hg19/GRCh37 (Feb 2009) proceeded in a stepwise manner, as described above, resulting in 101,622 reads (15.03% of input, or 50.7% of filtered reads), 78,236 reads (13.38% of raw input, or 48.2% of filtered reads) for datasets 1 and 2 respectively. Since these datasets comprised single end reads, further alignment rounds (as described above) yielded 67,201 reads (9.94% of input reads) and 51,210 reads (8.76% of input reads) respectively.

Reasons for exclusion of reads throughout these filtering steps include the lack of a valid match on the reference genome, the presence of trimmed reads that contain only vector sequence, as previously described, or the presence of reads that could not be unambiguously mapped to the reference genome [11].

Following alignment, reads were analysed to identify 1981 and 1789 unique ISs from data sets 1 and 2 respectively, giving an average IS read coverage of 33.9 (dataset 1) and 28.6 (dataset 2) (range 1 – 1789). Variability in read depth of individual ISs can be ascribed to either the repeated recovery of a specific IS due to PCR amplification bias during library preparation and/or clonal expansion of vector-transduced cell populations after integration [16]. It is likely that the average read depth observed reflects the clonal expansion of individual cells following an integration event, since the cell populations being analysed were grown in culture for up to 2 weeks following transduction.

### Comparison of Ub-ISAP with alternative methodology

In order to compare IS data generated from Ub-ISAP with data generated via alternative methods, we compared both the workflow and outcome of raw sequence read processing via Ub-ISAP with that obtained using the web based Vector Integration Site Analysis (VISA, https://visa.pharmacy.wsu.edu/bioinformatics/) using single end reads in dataset 1 and 2 (VISA is unable to process paired end reads). Use of VISA required uploading of sequence files in the FASTA format, and input of vector and TR sequences. By contrast, Ub-ISAP was able to process FASTQ files directly from the sequencer platform, with input of 5′ TR and 3′ primer sequences. In Ub-ISAP, trimming of 3′ primer sequences from TR-selected and trimmed reads allows for better alignment to the reference genome. VISA does not allow for input of these sequences for trimming. We are unable to ascertain whether VISA can align such reads, as VISA output contains final ISs identity but not intermediate alignment files for review.

Processing time for VISA approached 30 h for analysis of datasets 1 and 2, each of which contained approximately half a million reads, due largely to queuing prior to processing. This processing time could vary for other users since it is not possible to gain knowledge of the background computing environment while using a remote web service. By contrast, processing of these datasets using Ub-ISAP on a local computer with 32 GB RAM and i7-4770 CPU processor @ 3.40GHz X 8 took a maximum of 20 min. The process for calling of candidate retroviral integration sites (RIS) by VISA [16] generated 1576 and 1801 unique ISs from datasets 1 and 2 respectively, compared with 1981 and 1789 with Ub-ISAP. These differences are due to the filtering processes applied for each method (Table 2), with Ub-ISAP being more stringent with three progressive alignment rounds (as described above for single end reads), the requirement for a higher alignment score and percent identity, and a single specific query start site rather than allowing for a 3-bp variation. For unique ISs in dataset 1, Ub-ISAP and VISA assigned 547 and 738 gene symbols (respectively) of which 545 gene symbols were common. Similarly, for dataset 2, 409 gene symbols were commonly assigned by both Ub-ISAP and VISA, out of 455 and 617 total gene symbols respectively.

**Table 2:** Comparison of alignment criteria of Ub-ISAP and VISA

Alignment criteria	Ub-ISAP	VISA
Alignment score	100	>60
Percent identity	100	>92
IS distance from query start site (QSS)	0 bp	3 bp
Acceptance for IS calling	Reads aligning at more than one position are rejected	Top 5 candidates highest alignment score)

The greatest advantage of Ub-ISAP over VISA in our hands was the significant reduction in processing time in addition to the stringency of alignment calling, with a similar IS yield. Other advantages relating to the running of Ub-ISAP on the UNIX operating system on a local computer included the ability to customise Ub-ISAP for alternative IS selection criteria, and the possibility of further custom tool development. Another advantage of Ub-ISAP over VISA is the capacity to process paired end reads, which may provide greater certainty of mapping ISs within repetitive regions of the genome, and an overall greater confidence in the reliability of output ISs datasets, since only concordant reads from each end of a given junction fragment proceed to the alignment process.

### Comparison of Ub-ISAP output with published IS data

Ub-ISAP was further validated as an efficient IS analysis tool by re-analysis of two existing, independent sets of raw sequence reads, analysed using alternative custom workflows, for which published ISs data were available [1, 24]. Firstly, we re-analysed a data set with single end reads that was generated from DNA libraries prepared using human haematpoietic stem cells transduced with a clinical grade retroviral vector under differing culture conditions, designated Paris (P) or London (L) [24]. The P and L data sets with 8,706,511 and 7,503,456 raw reads (respectively) were analysed with Ub-ISAP to give 255,502 and 61,067 unique IS, marginally more than that recovered using the published methodology (250,215 and 54,424 for P and L datasets respectively, Table 3).

**Table 3:** Comparison of IS analysis of published data [24] for Paris (P) and London (L) samples with Ub-ISAP

Dataset	Total ISs	TSS-proximal	Intragenic	Intergenic
P-[24]	250,215	66,991 (26.77%)	108,033 (43.18%)	75,191 (30.05%)
P-Ub-ISAP	255,502	60,588 (24%)	110,101 (43%)	84,813 (33%)
L-[24]	54,424	12,538 (23.03%)	23,355 (42.91%)	18,532 (34.06%)
L-Ub-ISAP	61,067	12,269 (20%)	25,898 (42%)	22,900 (37%)

Annotation of the ISs extracted for the P and L datasets via Ub-ISAP into TSS-proximal, intragenic and intergenic location, followed by pair-wise statistical comparisons of IS distribution between the three categories upheld the statistically significant differences demonstrated by Hallwirth and colleagues [24] in relation to TSS-proximal ISs, with significantly higher clustering around the TSS in the P culture conditions (Fisher’s exact test, *P* < 0.0001) than the L conditions. Also in agreement with these published data, was the presence of significantly fewer ISs outside gene coding regions (intergenic) under the P conditions compared with the L conditions (Fisher’s exact test, *P* < 0.0001). In contrast to the published analysis for intergenic location, in which there was no statistically significant difference between the P and L culture conditions, the number of ISs identified by Ub-ISAP were, however, significantly different in favour of a greater number within gene coding regions (intragenic) under the P culture conditions compared with the alternative L conditions (Fisher’s exact test, *P* < 0.0022). This result further supports the hypothesis suggested by Hallwirth et al. that the P conditions for transduction may impart a higher risk of insertional mutagenesis than do the alternative L conditions. The small increase in number of ISs recovered and annotated using Ub-ISAP (2.11% and 11.99% increase in P and L respectively) was sufficient to resolve a statistically significant difference between the conditions for this category of IS location.

**Fig. 2:**
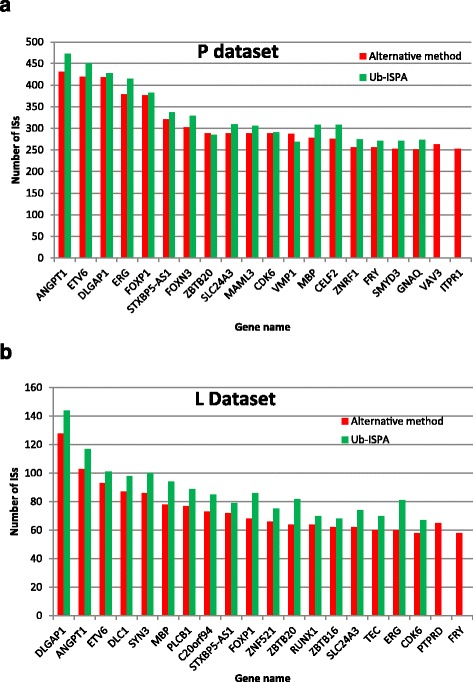
Comparison of the top 20 IS-containing genes identified from P and L datasets using Ub-ISAP and the alternative published methodology. **a**: Number of ISs (Y axis) for each of the top 20 IS containing genes (X axis) extracted from the unique ISs output derived from a published method (red columns) compared with Ub-ISAP (green columns) for the P dataset. **b** Number of ISs (Y axis) for each of the top 20 IS containing genes (X axis) extracted from the unique ISs output derived from a published method (red columns) compared with Ub-ISAP (green columns) for the L dataset

Since the above-mentioned comparison is limited to the total number of annotated ISs recovered after analysis rather than their identity, we also sought to compare the identity of ISs recovered using the two different pipelines by extracting data for the top 20 IS-containing genes for both the P and L datasets derived from each different method. This exercise demonstrated that Ub-ISAP extracted an identical list for the top 18/20 genes targeted for integration (starting with ANGPT1 and DLGAP1 for P and L respectively, Fig. 2) as that extracted by the published methodology, with (in general) a slightly higher number of ISs per targeted top 20 gene extracted by Ub-ISAP.

**Fig. 3:**
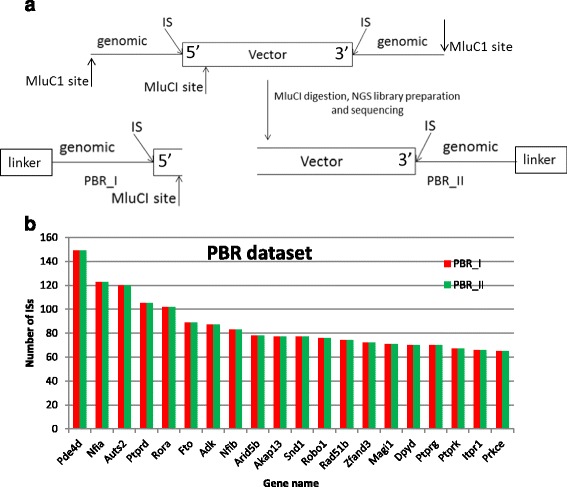
Generation and analysis of paired end reads from integrated AAV vector cassettes. **a** Diagrammatic representation of PBR_I and PBR_II DNA junction fragment library preparation. The integrated vector cassette and flanking genomic DNA was subjected to restriction enzyme digestion using MluC1, and PCR amplicons generated from both the resultant 5′ and 3′ ends of the vector cassette, using PBR_I and PBR_II primer sets (respectively) pooled prior to sequencing. Ub-ISAP was run separately to extract ISs using both primer sets from the same raw sequencing read file. **b** Comparison of the top 20 IS-containing genes (X axis) extracted from unique ISs output derived from sequential Ub-ISAP analysis of a pooled DNA fragment library prepared with alternative primer sets to derive the PBR_I and PBR_II datasets. Red columns show the number of ISs identified for each gene from the PBR_I data compared with the number identified from the PBR_II data (green columns)

The second published dataset against which we compared Ub-ISAP performance and outcome contained paired-end reads generated from a DNA library prepared using liver cells transduced with an adeno-associated virus (AAV)/piggyBac combination vector system that drives integration of an otherwise predominantly non-integrating AAV vector cassette into the liver cell genome in mice through a transposon mediated mechanism [1]. The library preparation method, involving restriction digestion of the gDNA of transduced cells and ligation of linkers, was similar to that used to generate libraries from retrovirally transduced cells, with an additional capacity to generate vector junction fragments bounding both the genomic/5′ vector cassette boundary and the 3′ vector cassette/genomic boundary of each IS (Fig. 3) using PCR. PCR amplicons representing both of these junction fragment types, designated PBR_I (reading into the 5′ genomic region) and PBR_II (reading into the 3’ genomic region) were pooled prior to the sequencing reaction. The 48,599,540 raw sequence read output file was analysed using Ub-ISAP to generate and classify 134,135 and 134,120 unique IS for PBR_I and PBR_II respectively, an increase of approx. 5% over that recovered using the published methodology (127,386). These analyses simply required separate runs through Ub-ISAP, following input of 5′ and 3′ paired primer sequences specific for each of the PBR_I or PBR_II generated junction fragments. Alignment of putative ISs was performed against the mm9 mouse genome. Annotation of the ISs extracted via Ub-ISAP for PBR_I and PBR_II into TSS-proximal (12%), intrageneic (51%) and intergenic (37%) locations were in line with the published data.

As data for extraction of the top 20 targeted genes for the published data were unavailable, we instead compared the top 20 targeted genes for integration between the PBR-I and PBR_II ISs generated by Ub-ISAP, predicting concordance between these lists, given that both ends of the same integrated vector cassette would have been represented within the pooled DNA junction fragment library that underwent sequencing. This exercise verified that the list of top 20 genes containing ISs was identical for PBR-I and PBR_II generated paired-end reads (Fig. 3) and in fact, genomic coordinates for the ISs between the PBR-I and PBR-II generated ISs were identical. Therefore, separate analyses of the same raw sequence read pool with Ub-ISAP, using alternative paired primer sequences for identification of putative ISs from each end of the integrated vector sequence was able to extract concordant data, in line with the underlying expectation based on the design of the library preparation system.

The parameters discussed above to filter the raw and the aligned reads to identify unique ISs using Ub-ISAP are pre-defined and recommended, but these could be changed by users, with basic knowledge of Unix scripting. The usage of dataset 1 and 2 from the Thermofisher PGM platform, datasets PBR_I and PBR_II from Illumina Mi-Seq and datasets P and L from Illumina Genome Analyser IIx (GAIIx), and the flexibility to change the parameters, proves the capability of Ub-ISAP to analyse datasets from varying NGS platforms. Ub-ISAP provides a concise result, with chromosomal positioning and closest gene accession number and symbol from the processed reads in tabular format as.txt file, and containing the unique ISs characterised as TSS-proximal, intragenic or intergenic. Being a UNIX-based tool, Ub-ISAP is designed to be installed on a local computer allowing shorter processing times. Another additional advantage for Ub-ISAP is that the reference genome database and version used for alignment (human or other) can be user-defined and can be easily updated. Ub-ISAP is therefore of utility across all species and into the future as additional genomic databases become available.

## Conclusion

Integration site (IS) analysis is an important step for assessing the safety and efficiency of gene therapies that use integrating viral vectors. Ub-ISAP is a time and memory efficient UNIX-based pipeline that allows researchers to analyse NGS datasets for ISs in a consistent manner. It can be readily updated with the current reference genome. Results are returned in a simple format to allow easy analysis of integration profiles of viral vectors.

## Abbreviations

AAV: Adeno-Associated Viral
gDNA: genomic DNA
GRCh37: Genome Reference Consortium Human build 37
GT: Gene therapy
IM: Insertional mutagenesis
IS: Integration site
L: London
NGS: Next-generation sequencing
P: Paris
PGM: Personal Genome Machine
RE: Restriction endonuclease
SCID-X1: X-linked severe combined immunodeficiency
TSS: Transcription start site
Ub-ISAP: UNIX-based vector integration site analysis pipeline
UCSC: University of California Santa Cruz

## References

[1] Cunningham SC, Siew S, Hallwirth CV, Bolitho C, Sasaki N, Garg G, Michael I, Hetherington N, Carpenter K, de Alencastro G, Nagy A, Alexander IE (2015). Modeling correction of severe urea cycle defects in the growing murine liver using a hybrid recombinant adeno-associated virus/piggyBac transposase gene delivery system. Hepatalogy.

[2] Beard BC, Adair JE, Trobridge GD, Kiem HP (2014). High throughput genomic mapping of vector integration sites in gene therapy studies. Methods Mol Bio.

[3] Trobridge GD (2011). Genotoxicity of retroviral hematopoietic stem cell gene therapy. Expert Opin Biol Ther.

[4] Boztug K, Schmidt M, Schwarzer A, Banerjee PP, Díez IA, Dewey RA, Böhm M, Nowrouzi A, Ball CR, Glimm H, Naundorf S, Kühlcke K, Blasczyk R, Kondratenko I, Maródi L, Orange JS, von Kalle C, Klein C (2010). Stem-cell gene therapy for the Wiskott-Aldrich syndrome. N Engl J Med.

[5] Aiuti A, Biasco L, Scaramuzza S, Ferrua F, Cicalese MP, Baricordi C, et al. Lentiviral hematopoietic stem cell gene therapy in patients with Wiskott-Aldrich syndrome. Science. 2013; doi:10.1126/science.1233151. 10.1126/science.1233151 PMC4375961 23845947

[6] Biffi A, Montini E, Lorioli L, Cesani M, Fumagalli F, Plati T, et al. Lentiviral hematopoietic stem cell gene therapy benefits metachromatic leukodystrophy. Science. 2013; doi:10.1126/science. 10.1126/science.1233158 23845948

[7] Bushman FD (2007). Retroviral integration and human gene therapy. J Clin Invest.

[8] Bushman F, Lewinski M, Ciuffi A, Barr S, Leipzig J, Hannenhalli S, Hoffmann C (2005). Genome-wide analysis of retroviral DNA integration. Nat Rev Microbiol.

[9] Naldini L (2011). Ex vivo gene transfer and correction for cell-based therapies. Nat Rev Genet.

[10] Gabriel R, Eckenberg R, Paruzynski A, Bartholomae CC, Nowrouzi A, Arens A, Howe SJ, Recchia A, Cattoglio C, Wang W, Faber K, Schwarzwaelder K, Kirsten R, Deichmann A, Ball CR, Balaggan KS, Yáñez-Muñoz RJ, Ali RR, Gaspar HB, Biasco L, Aiuti A, Cesana D, Montini E, Naldini L, Cohen - Haguenauer O, Mavilio F, Thrasher AJ, Glimm H, von Kalle C, Saurin W, Schmidt M (2009). Comprehensive genomic access to vector integration in clinical gene therapy. Nat Med.

[11] Calabria A, Leo S, Benedicenti F, Cesana D, Spinozzi G, Orsini M, et al. VISPA: a computational pipeline for the identification and analysis of genomic vector integration sites. Genome Med. 2014; 10.1186/s13073-014-0067-5 10.1186/s13073-014-0067-5 PMC4169225 25342980

[12] Ranzani M, Annunziato S, Adams DJ, Montini E (2013). Cancer gene discovery: exploiting insertional mutagenesis. Mol Cancer Res.

[13] Hayakawa J, Washington K, Uchida N, Phang O, Kang EM, Hsieh MM, et al. Long term vector integration site analysis following retroviral mediated gene transfer to hematopoietic stem cells for the treatment of HIV infection. PLoS One. 2009; doi:10.1371/journal.pone.0004211. 10.1371/journal.pone.0004211 PMC2615408 19148292

[14] Hacein-Bey-Abina S, Von Kalle C, Schmidt M, MP MC, Wulffraat N, Leboulch P, Lim A, Osborne CS, Pawliuk R, Morillon E, Sorensen R, Forster A, Fraser P, Cohen JI, de Saint Basile G, Alexander I, Wintergerst U, Frebourg T, Aurias A, Stoppa-Lyonnet D, Romana S, Radford-Weiss I, Gross F, Valensi F, Delabesse E, Macintyre E, Sigaux F, Soulier J, Leiva LE, Wissler M, Prinz C, Rabbitts TH, Le Deist F, Fischer A, Cavazzana-Calvo M (2003). LMO2-associated clonal T cell proliferation in two patients after gene therapy for SCID-X1. Science.

[15] Nam C, Rabbitts T (2006). The role of LMO2 in development and in T cell leukemia after chromosomal translocation or retroviral insertion. Molecular Ther.

[16] Hocum J, Battrell L, Maynard R, Adair J, Beard B, Rawlings D, et al. VISA - vector integration site analysis server: a web-based server to rapidly identify retroviral integration sites from next-generation sequencing. BMC Bioinformatics. 2015; doi:10.1186/s12859-015-0653-6. 10.1186/s12859-015-0653-6 PMC4493804 26150117

[17] Schinke EN, Bii V, Nalla A, Rae DT, Tedrick L, Meadows GG, et al. A novel approach to identify driver genes involved in androgen-independent prostate cancer. Mol Cancer. 2014; doi:10.1186/1476-4598-13-120. 10.1186/1476-4598-13-120 PMC4098713 24885513

[18] Hematti P, Hong BK, Ferguson C, Adler R, Hanawa H, SellersS, Holt IE, Eckfeldt CE, Sharma Y, Schmidt M, von Kalle C, Persons DA, Billings EM, Verfaillie CM, Nienhuis AW, Wolfsberg TG, Dunbar CE, Calmels B. Distinct genomic integration of MLV and SIV vectors in primate hematopoietic stem and progenitor cells. PLoS Biol. 2004;doi: 10.1371/journal.pbio.0020423. 10.1371/journal.pbio.0020423 PMC529319 15550989

[19] Brady T, Roth SL, Malani N, Wang GP, Berry CC, Leboulch P, et al. A method to sequence and quantify DNA integration for monitoring outcome in gene therapy. Nucleic Acids Res. 2011; doi:10.1093/nar/gkr140. 10.1093/nar/gkr140 PMC3113588 21415009

[20] Appelt J, Giordano F, Ecker M, Roeder I, Grund N, Wagenblatt A, Opelz G, Zeller W, Allgayer H, Fruehauf S, Laufs S (2009). QuickMap: a public tool for large-scale gene therapy vector insertion site mapping and analysis. Gene Ther.

[21] Kamboj A, Hallwirth CV, Kramer B. Ub-ISAP: a streamlined UNIX pipeline for mining unique viral vector integration sites from next generation sequencing data. Poster presented at: NGS 2017; Barcelona, Spain. 10.1186/s12859-017-1719-4 PMC5474025 28623888

[22] Ciuffi A, Ronen K, Brady T, Malani N, Wang G, Berry CC, Bushman FD (2009). Methods for integration site distribution analyses in animal cell genomes. Methods.

[23] Marcel M (2011). Cutadapt removes adapter sequences from high-throughput sequencing reads. EMBnet J.

[24] Hallwirth CV, Garg G, Peters T, Kramer B, Malani N, Hyman J, Ruan X, Ginn S, Hetherington N, Veeravalli L, Shahab A, Ranganathan S, Wei C, Liddle C, Thrasher A, Bushman F, Buckley M, Alexander IE (2015). Coherence analysis discriminates between retroviral integration patterns in CD34+ cells transduced under differing clinical trial conditions. Mol Ther Methods Clin Dev.

[25] Ambrosi A, Cattoglio C, Serio C. Retroviral Integration Process in the Human Genome: Is It Really Non-Random? A New Statistical Approach. PLoS Comput Biol. 2008;doi: 10.1371/journal.pcbi.1000144. 10.1371/journal.pcbi.1000144 PMC2453317 18688267

